# Nicorandil‐induced colovesical fistula in a patient with diverticular disease

**DOI:** 10.1002/ccr3.3888

**Published:** 2021-02-10

**Authors:** James D. Noyes, Ify R. Mordi, Qaiser Zeb, Chim C. Lang

**Affiliations:** ^1^ Division of Molecular & Clinical Medicine School of Medicine University of Dundee Dundee UK

## Abstract

Nicorandil's adverse effects can cause severe patient morbidity and can present to any specialty. Those with underlying diverticular disease are most susceptible. Medication reviews are vital for patients presenting with ulcer or fistula symptoms.

## Graphical abstract

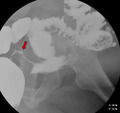

## 1 INTRODUCTION

Nicorandil, a commonly prescribed antianginal drug, has been linked to the development of gastrointestinal ulcers and fistulas. Its use is cautioned in diverticular disease; however, this is not apparent to many prescribers. Early identification of nicorandil's adverse drug effects coupled with prompt cessation of treatment can prevent significant patient harm.

The most recent figures show that there are 2.3 million people in the United Kingdom living with coronary heart disease.^1^ Nicorandil is recommended by the European Society of Cardiology for the treatment of chronic coronary syndromes as a second line therapy after beta blockers, calcium channel blockers, and long acting nitrates.^2^ It acts to dilate coronary resistance arterioles through the activation of adenosine triphosphate sensitive potassium channels and dilates both epicardial coronary arteries alongside systemic veins through nitric oxide donation. These actions result in increased coronary blood flow with a reduction of both preload and afterload.^3^ The off‐target side effects of this therapy can have major consequences to patients, especially those with diverticular disease.

The term “diverticular disease” incorporates both the presence of diverticula and diverticulitis. The majority of people aged 60 and over in Western populations have diverticular disease which is often asymptomatic. Therefore, the potential for nicorandil to cause widespread harm to patients is high.^4^ Recognizing patients who are experiencing nicorandil's side effects cannot be limited to one speciality. The adverse effects can be wide ranging and ulcers can occur at any skin or mucosal site; including the eyes and oral cavity.^5^ Therefore, it is important to widely educate healthcare professionals to identify these signs so that nicorandil can be promptly stopped and alternatives offered by a cardiologist. In addition to reducing significant patient burden, cardiovascular therapies are major contributors to the NHS’ annual £466 million bill from adverse drug reaction related hospital admissions.^6^

This case report follows a 61‐year‐old gentleman who was prescribed nicorandil for his stable angina in the background of CT‐proven diverticular disease. Two years after initiation of nicorandil he developed a colovesical fistula which resulted in numerous hospital admissions and major surgery. He remained on nicorandil for seven years post fistula diagnosis. Through highlighting this case, we aim to reduce future patient morbidity caused by nicorandil's adverse effects.

## 2 CASE HISTORY

The patient was first prescribed nicorandil in 2010 to treat recurrent episodes of chest pain, resistant to first‐line therapies, which were not associated with elevated troponin levels or abnormalities on coronary angiography. His past medical history included two myocardial infarctions. In 2012, the patient presented to his general practitioner (GP) with a five‐day history of increased urinary frequency and a clear discharge after micturition. Urinalysis revealed leucocytes, nitrites, blood, and protein. He was commenced empirically on a seven‐day course of trimethoprim; urine culture results showed a mixed growth of organisms. His symptoms did not improve with antibiotic therapy and he quickly represented to his GP. Further questioning at this time revealed he was passing air at the end of micturition for up to 10 seconds. Additionally, he reported the presence of “tissue” in his urine which was dark in color. There were no reports of frank hematuria or fever. On examination, vital signs were normal, and abdominal examination revealed suprapubic tenderness radiating to his left iliac fossa. An urgent follow‐up by the colorectal team was requested.

## 3 INVESTIGATIONS

**FIGURE 1:**
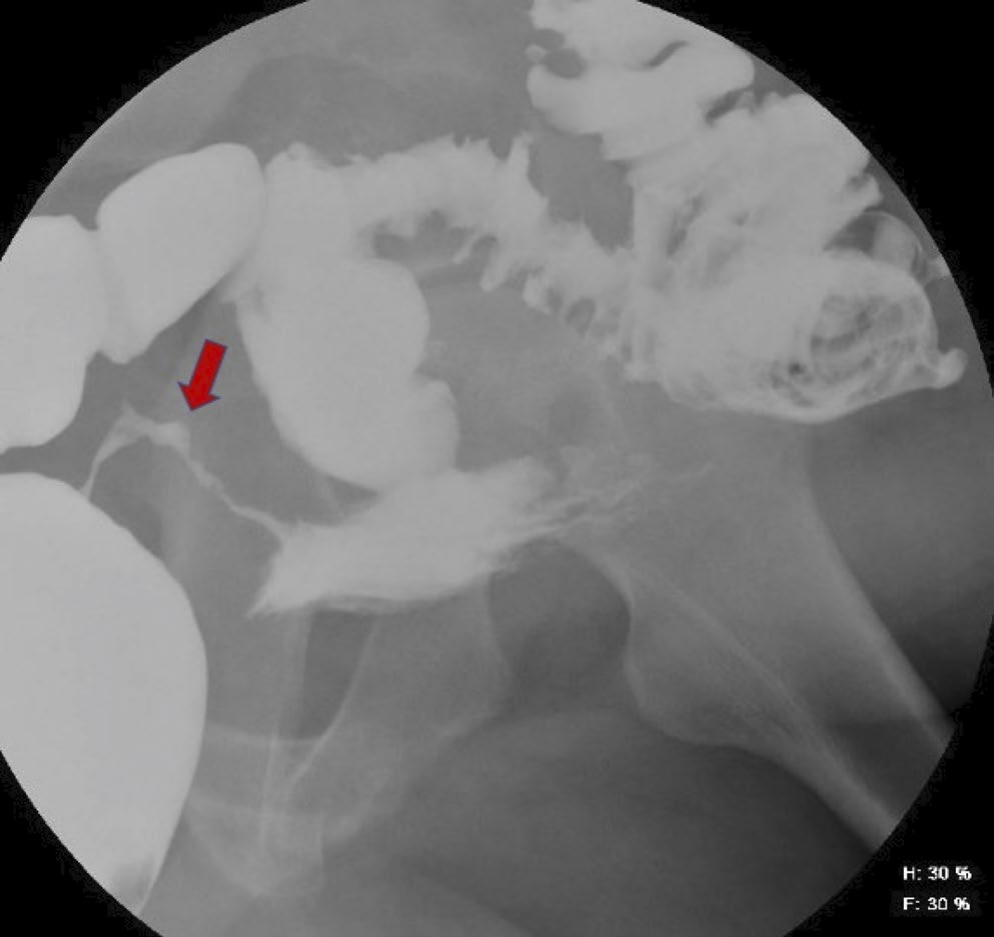
Barium enema highlighting the fistula between the rectum and bladder

The colorectal team carried out a CT scan of his abdomen and pelvis, colonoscopy, and barium enema. The CT scan showed a channel of communication from the mid‐rectum into the posterior bladder. Gas locules were visualized in the urinary bladder alongside a trace of rectal contrast. Barium enema showed a leakage of barium from the rectum anteriorly passing anteriorly to fill an irregular cavity (Figure 1). Colonoscopy findings excluded any malignant disease.

The CT scan was compared with a previous abdominal CT scan carried out three years earlier, undertaken due to an acute episode of abdominal pain which was attributed to a paracolic abscess around an area of diverticular disease of the sigmoid colon. The updated CT scan showed that the identified communication was a new finding and the previously observed paracolic abscess had completely resolved. Due to the location of the fistula, a defunctioning ileostomy was offered to the patient which he declined at the time due to his anxiety about having surgery.

In 2017, he re‐presented with urosepsis and a further abdominal CT scan showed an additional fistula had formed. The second colovesical fistula was located between a diverticulum and the anterior superior bladder; the previously identified fistula, located at the posterior bladder, was still patent.

## 4 DIFFERENTIAL DIAGNOSIS

The clinical presentation and examination findings strongly suggested that the patient had an abnormal connection between his colon and bladder. Investigations were essential for two reasons: firstly, to understand the anatomy of this suspected abnormal connection and secondly to establish its cause.

A number of different pathologies can result in colovesical fistula formation. The most important to exclude was malignancy. The clinical history was not suggestive of this as the patient reported no change in bowel habits, weight loss or family history of malignancy. However, colonoscopy was essential to definitively exclude this. Findings from this examination were also able to eliminate inflammatory bowel disease as an underlying cause of his fistula.

The CT scan findings showed significant diverticular disease of the sigmoid colon, which in the absence of other pathology appeared the most likely cause of the fistula. The potential role that nicorandil played in this process was not initially considered.

## 5 TREATMENT

To prevent further episodes of urosepsis, surgical treatment was required to remove the diseased colon containing diverticula. The patient underwent an elective Hartmann's procedure with end colostomy in 2018. Intraoperative findings revealed the sigmoid colon was adhered to the posterior aspect of the bladder. The sigmoid colon was dissected from the bladder and removed, leaving a healthy rectal stump in situ.

**FIGURE 2:**
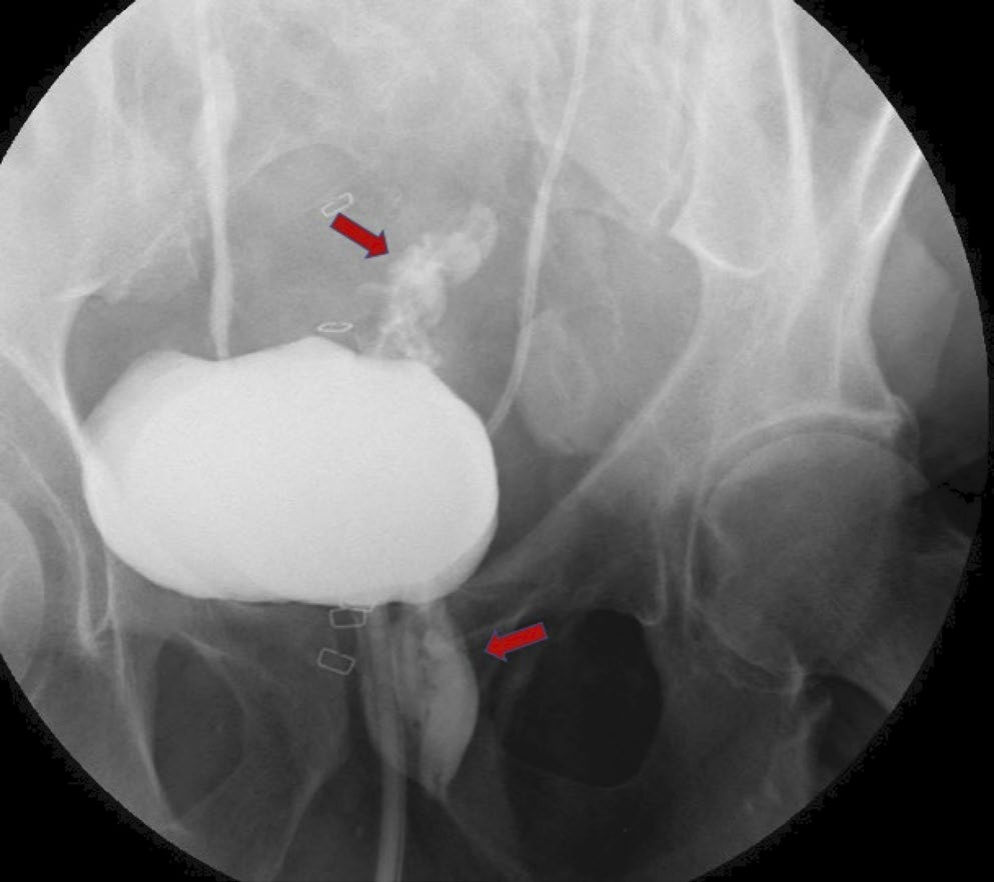
Cystogram highlighting the leakage of contrast from the bladder into the bowel

Postoperatively, blood was observed in his catheter bag and urine was noted to be leaking from the patient's rectal stump. This was investigated with a cystogram which showed evidence of contrast leaking from the posterior bladder into bowel (Figure 2). A repeat CT scan revealed a persistent fistula located in the posterior bladder connecting to the defunctioned rectal stump. The patient received a further course of antibiotics to treat a proven urinary tract infection prior to his hospital discharge. He represented two weeks later with a wound infection and wound dehiscence, requiring emergency surgical intervention. At this time a urological opinion was sought and consideration was given to long‐term catheter use.

## 6 OUTCOME AND FOLLOW‐UP

Subsequent clinic review by the colorectal team found that he was still symptomatic, suggesting the fistula was not healing despite catheter use. His symptoms were most severe when the catheter became blocked. In light of this, the decision was taken to make the catheter permanent and to have regular catheter changes scheduled in the community. The psychological burden of his ongoing symptoms had a large negative impact on his quality of life and there were periods of time when the patient felt unable to leave his home.

Shortly after the Hartmann's procedure, he was routinely reviewed by the cardiology team. At this time the association between the patient's fistula and nicorandil use was noted, seven years after the fistula was first diagnosed. The cardiology team stopped the nicorandil and started the patient on an alternative antianginal therapy, ranolazine. The patient's anginal symptoms have since been well controlled on this new therapy.

No further interventions regarding his fistula are currently planned as he has recently received a new diagnosis, unrelated to the above history, of a squamous cell carcinoma with extensive cervical nodal metastases.

## 7 DISCUSSION

This case demonstrates the significant morbidity that diverticular disease can cause patients when complications develop. While fistula formation is a known consequence of diverticular disease in the absence of nicorandil, evidence shows that patients with diverticular disease on nicorandil therapy have a 7‐fold increased risk of fistula formation compared to those not taking the drug.^7^ Previous case series have also highlighted nicorandil's link to fistulation between organs.^8^ Despite published evidence and the British National Formulary's caution, this case shows that nicorandil continues to be prescribed to at‐risk patients. The case also illustrates the challenge of promptly identifying nicorandil's adverse effects as patients rarely present back to cardiology with their symptoms. It is far more common for patients to seek help from others, such as general practitioners, dermatologists or surgeons who may be less familiar with nicorandil.^9^

The pathophysiology of nicorandil‐induced ulceration and fistulation is yet to be determined. The most probable mechanism involves the accumulation of nicorandil's metabolites, nicotinic acid, and nicotinamide. In normal circumstance, these combine to form a pool of nicotinamide adenine dinucleotide which is found widely in organisms and causes no harm. In certain people who suffer from adverse effects, it is postulated that this pool becomes saturated leading to the accumulation of the individual toxic metabolites.

Nicotinamide then increases blood flow at the edge of a vulnerable area which stimulates epithelial proliferation. Nicotinic acid subsequently ulcerates this epithelial layer and floods the remaining vulnerable tissue.^10^ In the colon, diverticula are formed at points of blood vessel herniation through the muscle wall creating vulnerable tissue with a rich vascular supply. Nicorandil‐induced fistulation has also been hypothesized to occur as a result of the release of pro‐inflammatory nitric oxide in the sigmoid mucosa.^7^

Local prescribing guidance for stable angina, based on Scottish Intercollegiate Network Guideline (SIGN) 96 and National Institute for Health and Care Excellence (NICE) Clinical Guideline 126, recommends adding nicorandil after a beta blocker or/and a calcium channel blocker and isosorbide mononitrate. Within our health board, nicorandil can be started by primary care doctors without cardiology consultation. The local protocol specifically states nicorandil should “not be used in patients with prior or risk factors for gastrointestinal ulceration.”^11^ Further treatments, such as ranolazine, can only be prescribed with permission of a consultant cardiologist in our region with initial prescriptions coming from the main hospital pharmacy. Prior to this, requests had to be granted through individual patient treatment requests to the Scottish Medicines Consortium.

A 10‐year observational study of French nicorandil‐induced ulceration cases concluded that their development could not be predicted.^10^ Evidence has been discussed showing that individuals with known diverticular disease are at greater risk of complications and we have demonstrated the burden of this at a human level through our case. However, many individuals have clinically silent diverticular disease which presents both a risk to patients and a challenge to cardiologists.

Diverticulosis is often detected during imaging for other reasons and there is currently no indication to monitor these individuals further as a result of the finding.^12^ Before starting nicorandil therapy, patients do not routinely undergo investigations to look for the presence of diverticula. Current methods of identifying diverticular disease include CT scanning and colonoscopy, both of which are expensive and have risks. The aetiology of diverticular disease has shifted latterly with advances in genetic studies revealing 48 associated loci.^13^ Epidemiological studies have predicted the heritability of diverticular disease to be up to 50% with an increased prevalence of diverticula noted in monozygotic co‐twins compared to dizygotic co‐twins.^12^, ^14^ Advances in pharmacogenetics have shown that people with a high genetic risk of diverticular disease are more likely to stop nicorandil therapy early.^15^ This provides an insight to future strategies aimed at tailoring antianginal treatment based on individual patient risk of adverse drug effects.

The pharmacogenomic advances are an exciting prospect that will hopefully reduce the incidence of future ulcers and fistulas in years to come. Today, the focus must remain on educating patients prescribed nicorandil and all healthcare professionals to the risk of nicorandil's off‐target side effects. Nicorandil is prescribed for symptom control of angina and shows no long‐term benefit to survival. When side effects are suspected it is important to stop nicorandil therapy promptly to limit patient harm. Discussions with cardiology regarding an alternative antianginal should not delay cessation of nicorandil.^9^ A surgical case series has shown that stopping nicorandil resulted in improved healing of anal ulcers; whereas all surgical interventions to treat the ulcers were unsuccessful.^16^ Additionally, stopping nicorandil was also shown to promote the healing of previously non‐healing surgical wounds in another surgical case series.^17^

Our case highlights the importance of all healthcare professionals performing a thorough medication review when a patient presents with new symptoms. For those who prescribe nicorandil, it is imperative to consider the high prevalence of diverticular disease in the Western world today and to inform patients of nicorandil's adverse effects when prescribed. Many patients requiring antianginals will have had previous abdominal imaging performed; reviewing this information and identifying those at risk of side effects can allow for alternative antianginal therapies to be offered.

## CONFLICT OF INTEREST

All authors declared no competing interests for this work.

## AUTHOR CONTRIBUTIONS

JDN: researched the case and wrote the manuscript. IRM and QZ: contributed to the manuscript and following revisions. CCL: involved in the clinical care of the patient and supervised the writing of the manuscript. All authors read and approved the final manuscript.

## ETHICAL APPROVAL

Patient consent was gained and no further ethical approval was required.

## Data Availability

Data sharing not applicable to this article as no datasets were generated or analysed during the current study.
